# Differential gene expression associated with postnatal equine articular cartilage maturation

**DOI:** 10.1186/1471-2474-9-149

**Published:** 2008-11-05

**Authors:** Michael J Mienaltowski, Liping Huang, Arnold J Stromberg, James N MacLeod

**Affiliations:** 1 University of Kentucky, Department of Veterinary Science, M.H. Gluck Equine Research Center, Lexington, KY, 40546-0099, USA; 2 University of Kentucky, Department of Statistics, 867 Patterson Office Tower, Lexington, KY, 40506-0027, USA

## Abstract

**Background:**

Articular cartilage undergoes an important maturation process from neonate to adult that is reflected by alterations in matrix protein organization and increased heterogeneity of chondrocyte morphology. In the horse, these changes are influenced by exercise during the first five months of postnatal life. Transcriptional profiling was used to evaluate changes in articular chondrocyte gene expression during postnatal growth and development.

**Methods:**

Total RNA was isolated from the articular cartilage of neonatal (0–10 days) and adult (4–5 years) horses, subjected to one round of linear RNA amplification, and then applied to a 9,367-element equine-specific cDNA microarray. Comparisons were made with a dye-swap experimental design. Microarray results for selected genes (COL2A1, COMP, P4HA1, TGFB1, TGFBR3, TNC) were validated by quantitative polymerase chain reaction (qPCR).

**Results:**

Fifty-six probe sets, which represent 45 gene products, were up-regulated (p < 0.01) in chondrocytes of neonatal articular cartilage relative to chondrocytes of adult articular cartilage. Conversely, 586 probe sets, which represent 499 gene products, were up-regulated (p < 0.01) in chondrocytes of adult articular cartilage relative to chondrocytes of neonatal articular cartilage. Collagens, matrix-modifying enzymes, and provisional matrix non-collagenous proteins were expressed at higher levels in the articular cartilage of newborn foals. Those genes with increased mRNA abundance in adult chondrocytes included leucine-rich small proteoglycans, matrix assembly, and cartilage maintenance proteins.

**Conclusion:**

Differential expression of genes encoding matrix proteins and matrix-modifying enzymes between neonates and adults reflect a cellular maturation process in articular chondrocytes. Up-regulated transcripts in neonatal cartilage are consistent with growth and expansion of the articular surface. Expression patterns in mature articular cartilage indicate a transition from growth to homeostasis, and tissue function related to withstanding shear and weight-bearing stresses.

## Background

At birth, articular cartilage is relatively homogeneous. That is, the cellular and molecular structures do not vary from site to site topologically across the joint [1-6]. During postnatal maturation in mammals, articular cartilage undergoes architectural reorganization associated with growth and changes in biomechanical demands on the tissue [1-3,5]. For equine articular chondrocytes, the process is characterized by altered matrix protein production patterns with exercise during the first five months of postnatal life [2,7]. This adaptation affects the tissue longitudinally from the joint surface to deeper layers, as well as topologically across a joint between weight-bearing and non-weight-bearing areas [1,2,8]. Sites that receive low-level constant weight-bearing load have higher amounts of glycosaminoglycan and proteoglycan, providing resistance to compressive stresses [1,8]. Sites that receive high sheer stress through intermittent peak loading and movement have high levels of type II collagen for tensile strength [1,8]. As noted, these changes in architectural heterogeneity develop in the articular cartilage of foals during the first 5 months of life [2,3]. However, if exercise is restricted, articular cartilage fails to fully develop these characteristics of maturation. In addition, re-establishing normal levels of activity in a previously exercise-restricted foal is ineffective at promoting proper articular cartilage adaptation [2,3]. In this same population of horses, van den Hoogen *et al*. found that proteoglycan synthesis increased with free pasture exercise over the first 5 months followed by a light exercise regimen [9]. These results suggest that the architecture of articular cartilage changes with age and mechanical stimulation. If normal exercise is severely restricted in a horse during the first 5 months of postnatal life, then adaptation of matrix and anchorage proteins in articular cartilage to weight- and shear-stresses can be permanently compromised.

Postnatal articular cartilage changes have been characterized in several mammals. The transition from isotropic to anisotropic architecture was demonstrated in the articular cartilage of the proximal tibia of rats by Sasano *et al*. and in the medial femoral condyle of rabbits by Hunziker *et al*. [5,10]. Increased postnatal matrix deposition of type II collagen occurs in the rabbit and rat [10,11]. Changes and topological differences in cross-linking of collagens with age in postnatal horses as well as in humans have been established [7,8,12]. Moreover, moderate exercise in young adult dogs, guinea pigs, and humans seem to cause zonal changes in proteoglycan and collagen content that allow for adaptation and strengthening of articular cartilage [13]. While past studies have described biochemical and histological changes to articular cartilage during the postnatal period, much remains unexplained with regards to the cellular mechanisms that achieve growth and matrix re-organization. The current study utilizes microarray-based transcriptional profiling to assess differential gene expression between neonatal and mature articular cartilage across a large subset of genes in the equine genome. In addition to individual genes, including those with established functional annotation in cartilage, the approach enables expression differences between gene ontology categories to be compared. The hypothesis tested was that changes in chondrocyte gene expression occur during postnatal maturation and reflect a functional transition from growth and matrix synthesis to cartilage homeostasis.

## Materials and methods

### Cartilage Collection and RNA Isolation

Full-thickness articular cartilage was harvested and pooled from the proximal and distal humerus, the proximal and distal femur, the proximal and distal tibia, proximal radius, and proximal ulna of seven 0–10 day old foals and nine 4–5 year old adults. In the foals, particular attention was paid to distinguish regions with and without cartilage canals because articular cartilage with vasculature is destined to become calcified cartilage or to undergo hypertrophic differentiation and the formation of bone. Only cartilage without vascular canals was utilized in this study. Cartilage was rinsed in sterile phosphate-buffered saline, snap-frozen in liquid nitrogen, and then pulverized into powder while still frozen. Total RNA was isolated in a buffer of 4 M guanidinium isothiocyanate, 0.1 M Tris-HCl, 25 mM EDTA (pH 7.5) with 1% (v/v) 2-mercaptoethanol, followed by differential alcohol and salt precipitations [14-16]. Finally, RNA samples were re-purified using QIAGEN RNeasy columns following the manufacturer's protocol.

### Transcriptional Profiling

Microarray slides were printed with clones selected from a cDNA library generated using mRNA purified from the articular cartilage of a 15-month old Thoroughbred male [17]. Creation of the cDNA library is described in Coleman *et al*. [18]. Probe sets on the microarray slides were prepared and printed as described by Band *et al*. [19]. Transcriptional profile differences of neonatal and mature articular chondrocytes were assessed by four separate neonate-adult comparisons. For each experimental sample, 2 μg of total RNA received one round of linear amplification primed with oligo-dT (Invitrogen – SuperScript RNA Amplification System) [20,21]. Amplified RNA (2 μg) was then used as template to create fluorescent dye-coupled single-stranded aminoallyl-cDNA probes (Invitrogen – Superscript Indirect cDNA Labeling System, Molecular Probes – Alexa Fluor 555 and 647 Reactive Dyes).

Microarray slides were pre-hybridized in 20% formamide, 5× Denhardt's, 6× SSC, 0.1% SDS, and 25 μg/ml tRNA for 45 minutes as described by Band *et al*. [19]. Slides were then washed five times in deionized water and once in isopropanol and spun dry at 700 g for 3 minutes [19]. Two dye-coupled fluorescent cDNA probes representing one of each of the two experimental groups (neonate vs. adult) were combined with 1× hybridization buffer (1× Slide Hybridization Buffer #1, Ambion, cat no. 8801), incubated for 2 minutes at 95°C, and then applied to the slide under a glass lifterslip for 48 hours at 42°C. All hybridizations were performed in duplicate with a dye swap to eliminate possible dye bias [22]. Post-hybridization washes were each for 5 minutes as follows: first wash – 1× SSC, 0.2% SDS, 0.1 mM DTT at 42°C; second wash – 0.1× SSC, 0.2% SDS, 0.1 mM DTT at room temperature; third and fourth washes – 0.1× SSC and 0.1 mM DTT at room temperature. After the post-hybridization washes, the slides were spun dry under argon gas at 700 g for 3 minutes. Each slide was coated once in DyeSaver 2 (Genisphere) and allowed to dry for 10 minutes. Scanning was performed using a GenePix 4100A scanner with the spot intensities computed using GENEPIX 6.0 image analysis software (Axon Instruments/Molecular Devices).

### Statistics and Analysis

After scanning, median intensities adjusted for background for each pair of spots were lowess-normalized for every slide using the bottom row of each of the 48 printing blocks. This bottom row contains a constant set of positive and negative controls. A one sample t-test was applied to the log ratios accounting for dye-swapped slides. Corresponding microarray IDs for statistically significant probe sets were queried with an annotation database [23]. The data extracted were putative gene symbols and gene descriptions from a series of BLAST searches of the ESTs to the curated protein, RNA, and genomic Reference Sequence Databases [24]. Gene ontology (GO) annotation was derived from batch queries of the DAVID Bioinformatics tool or manually through individual NCBI Entrez Gene queries [25,26]. Statistical data, fold change quantities, and GO annotations were managed within an Excel spreadsheet (Microsoft, Redmond, WA). Expression patterns were analyzed using Expression Analysis Systematic Explorer (EASE) standalone software [27]. Microarray data were submitted to NCBI Gene Expression Omnibus as the data series GSE10303 for platform GPL6423 with sample accessions GSM260356, GSM260358, GSM260361, and GSM260363 [28].

### Validation of Microarray Hybridization Results with qPCR

Differential expression for selected genes was validated via quantitative polymerase chain reactions (qPCR). Briefly, total RNA was reverse-transcribed (RT) into cDNA using an oligo-dT primer with the Promega Reverse Transcription System (Promega, cat. no. A3500). Quantitative "real-time" PCR (ABI Systems 7900HT Fast Real-Time PCR System, Applied Biosystems, Foster City, CA) was performed using TaqMan Gene Expression Master Mix (Applied Biosystems, cat. no. 4369016) and intron-spanning primer/probe sets (Assays-by-Design, Applied Biosystems) created from equine genomic sequence data (Ensembl – ; UCSC Genome Browser – ) (Table 1). Each primer/probe set was tested for specificity and performance and had to meet two criteria: (1) no amplification with genomic DNA, minus-RT, and water controls; and (2) amplification efficiencies within a range of 0.9–1.2 as determined by manual serial dilution of a positive control sample, following Applied Biosystems recommended performance guidelines. All primer/probe sets met these specificity and performance criteria. Beta-2-microglobulin (B2M), beta-glucuronidase (GUSB), phosphoglycerate kinase 1 (PGK1), and large ribosomal protein P0 (RPLP0) primer/probe sets were evaluated as potential endogenous control transcripts. Steady state levels of mRNA encoding type II procollagen (COL2A1), cartilage oligomeric matrix protein (COMP), proline 4-hydroxylase (P4HA1), transforming growth factor beta receptor-3 (TGFβR3), and tenascin-C (TNC) were selected for RT-qPCR validation based on EASE data analyses. Transforming growth factor beta-1 (TGFβ1) was selected as a transcript of biological interest that did not demonstrate an expression difference by microarray hybridizations. Results were adjusted for efficiency as measured by LinRegPCR using the default fit option which measures the slope of a line containing 4–6 data points and the highest R^2 ^correlation value [29,30]. Real-time PCR data were normalized with endogenous control genes GUSB and RPLP0 according to guidelines set by geNorm stability analyses [31]. Relative expression levels by RT-qPCR using all 10 equine-specific primer/probe sets were determined individually on 7 neonatal and 9 adult articular cartilage RNA samples. This included the original 4 neonatal and 4 adult articular cartilage RNA samples used in the microarray comparisons. A non-parametric Mann-Whitney-Wilcoxon Rank Sum Test was used to assess statistical significance of qPCR results.

**Table 1:** Primer nucleotide sequences used in RT-qPCR assays for genes described in the study

**Gene Name**	**Gene Symbol**	**Forward Primer**	**Reverse Primer**
Beta-2-microglobulin	B2M	5-CGGGCTACTCTCCCTGACT-3	5-GTGACGTGAGTAAACCTGAACCTT-3

Beta-glucuronidase	GUSB	5-GCTCATCTGGAACTTTGCTGATTTT-3	5-CTGACGAGTGAAGATCCCCTTTT-3

Phosphoglycerate kinase-1	PGK1	5-GTGGCCTCTGGCATACCT-3	5-CTGCTCTCAGTGCCACAGT-3

Ribosomal protein, large, P0	RPLP0	5-CTGATTACACCTTCCCACTTGCT-3	5-AGCCACAAATGCAGATGGATCA-3

Procollagen, type II, alpha 1	COL2A1	5-CTGGCTTCAAAGGCGAACAAG-3	5-GCACCTCTTTTGCCTTCTTCAC-3

Cartilage oligomeric matrix protein	COMP	5-CGAGCCCGGCATCCA-3	5-CCCAGGGCCTGTGGAG-3

Proline-4-hydroxylase, alpha 1	P4HA1	5-CCTGTCCAGTGCTAGTAGGAAAC-3	5-CCTGTCCAGTGCTAGTAGGAAAC-3

Transforming growth factor beta-1	TGFβ1	5-CCCTGCCCCTACATTTGGA-3	5-TGTACAGGGCCAGGACCTT-3

Transforming growth factor beta receptor 3	TGFβR3	5-ACATCATCGAGTTAATCACCCCAAA-3	5-GATCCTTTCGAGAAGGTCTTATGTCA-3

Tenascin-C	TNC	5-TCAGCCATCACTACCAAGTTCAC-3	5-GAACCTCAGTAGCAGTCAAATCTCT-3

## Results

### Global assessment of transcriptome differences

**Figure 1:**
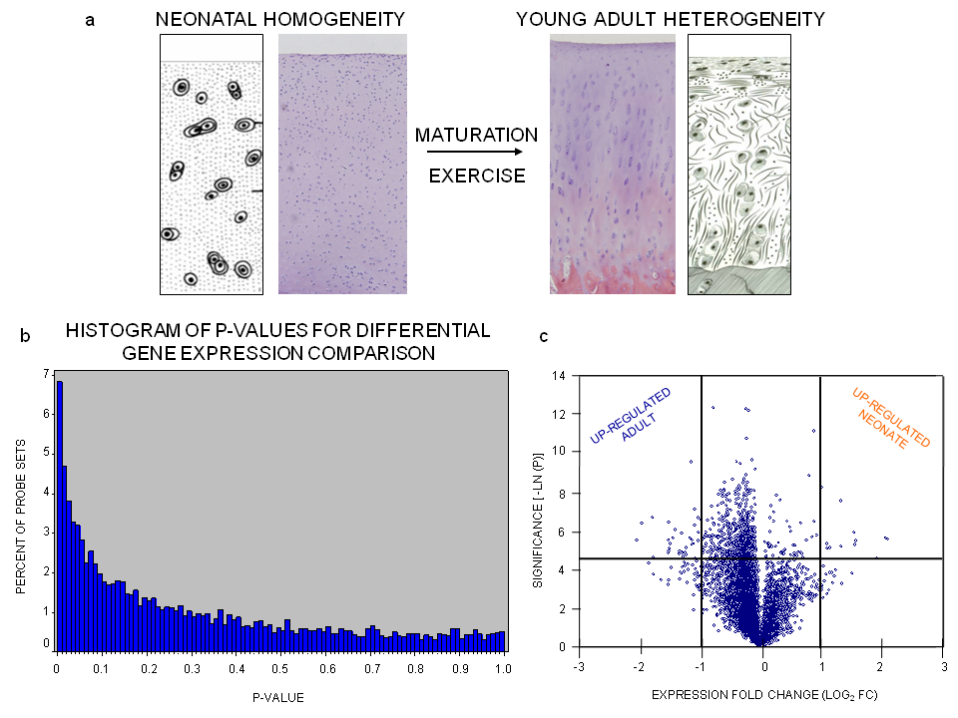
**cDNA microarray transcriptional profiling indicates differential gene expression between neonatal and adult articular cartilage**. (a) Histomorphometric comparison of isotropic articular cartilage structure in the equine neonate and the anisotropic structure in adults. (b) A p-value histogram of differentially expressed transcripts from the 9,367 element equine-specific cDNA microarray demonstrates that just under 7% of the clones had significance at p < 0.01. (c) A volcano plot of the neonatal versus adult comparison; points above the horizontal black line are transcripts demonstrating differential expression with a significance of p < 0.01. The two vertical black lines correspond to 2-fold up- and down-regulation. Degree of statistical significance is displayed along the vertical axis and expression fold change along the horizontal axis.

Nine thousand three hundred sixty-seven probe sets were analyzed. A total of 642 probe sets (6.9%) were differentially expressed (p < 0.01; Figure 1b). A volcano plot shows that 586 probe sets (representing 499 different genes) were up-regulated in adult chondrocytes relative to neonates, while 56 probe sets (representing 45 different genes) of the differentially expressed clones were up-regulated in neonate chondrocytes relative to adults (Figure 1c). Based on a p-value of 0.01, we would expect up to 94 (1% of 9,367) to be differentially expressed by chance, yielding a False Discovery Rate (FDR) of 14.64% (94/642).

### Functional patterns of differential gene expression

Significant genes with a > 2 fold difference in either direction (neonate > adult, adult > neonate) were analyzed by Expression Analysis Systematic Explorer (EASE) (Table 2).

**Table 2:** EASE analysis of genes with significant fold differences greater than 2

**GO System**	**Gene Category**	**List Hits**	**List Total**	**Population Hits**	**Population Total**	**EASE Score**	**Official Gene Symbols**
Cellular Component	Extracellular	19	37	258	2760	3.27E-10	CHAD, CILP, CLU, COL2A1, COL9A1, COMP, CP, FMOD, FN1, FRZB, GAS6, LOXL2, NID2, PRELP, SERPINA5, SULF1, THSD1, TIMP2, TNC

Cellular Component	Extracellular Matrix	11	37	101	2760	3.17E-07	CHAD, COL2A1, COL9A1, COMP, FMOD, FN1, NID2, PRELP, THSD1, TIMP2, TNC

Molecular Function	Extracellular Matrix Structural Constituent	6	40	35	2830	9.16E-05	CHAD, COL2A1, COL9A1, COMP, FN1, PRELP

Biological Process	Skeletal Development	6	38	55	2780	0.00068	ANKH, COL2A1, COMP, DLX5, FRZB, PRELP

Biological Process	Cell Adhesion	8	38	151	2780	0.00315	COL2A1, COL9A1, COMP, FN1, LOXL2, NID2, THSD1, TNC

Cellular Component	Extracellular Space	5	37	71	2760	0.01273	CP, FN1, LOXL2, SERPINA5, SULF1

Biological Process	Development	11	38	410	2780	0.03681	ANKH, BOC, CLU, COL2A1, COL9A1, COMP, DLX5, FRZB, LOXL2, PRELP, TGFBR3

Biological Process	Heparan Sulfate Proteoglycan Metabolism	2	38	5	2780	0.06484	HS3ST3A1, SULF1

Biological Process	Acute-phase Response	2	38	6	2780	0.07731	FN1, SERPINA5

Molecular Function	Scavenger Receptor Activity	2	40	6	2830	0.07996	KIAA1822, LOXL2

Biological Process	Organogenesis	7	38	231	2780	0.08131	ANKH, COL2A1, COL9A1, FRZB, PRELP

Molecular Function	Transmembrane Receptor Activity	5	40	122	2830	0.08470	FRZB, KIAA1822, LOXL2, PDGFRL, TNC

Molecular Function	Receptor Activity	7	40	233	2830	0.09626	FRZB, KIAA1822, LOXL2, NR1D2, PDGFRL, TGFBR3, TNC

"GO System" represents the major ontological system (i.e., cellular component, biological process, or molecular function). "Gene category" is a descriptive term shared by a group of genes. "List hits" are the numbers of differentially abundant transcripts that belong to the gene category. "List total" is the number of differentially expressed genes within the corresponding cellular component, biological process, or molecular function system. "Population hits" represent the total number of genes found on the microarray possessing that specific gene category annotation (e.g., extracellular, skeletal development, etc.). "Population Total" represents the total number of genes found on the microarray possessing that GO system annotation. "EASE score" is a measure of overrepresentation that scales the results of a statistical analysis (Fisher's exact test) by biasing against categories supported by few genes. Gene symbols of differentially expressed probe sets represented for each gene category are given in the last column.

Overrepresentation was determined by EASE score, a Fisher's exact test measure. Gene overrepresentation was strongest for extracellular and extracellular matrix categories (Table 2). Differential expression was also represented in categories involving skeletal development, cell adhesion, heparan sulfate proteoglycan metabolism, as well as receptor activity and organogenesis. Categories not overrepresented in this analysis, however, include growth factor activity and cell proliferation.

### Cartilage matrix proteins and modifying enzymes

Many of the differentially expressed transcripts identified in the annotation categories are cartilage matrix proteins. Collagen types II (COL2A1), and IX (COL9A1, COL9A2, COL9A3), and tenascin-C (TNC) were upregulated in neonatal chondrocytes relative to adult chondrocytes (Table 3). Other genes demonstrating greater transcript abundance in neonates relative to adults include collagen-modifying enzymes like lysyl oxidase-like 2 (LOXL2), proline-4-hydroxylase alpha 1 subunit (P4HA1), and the basement membrane protein nidogen-2 (NID2) (Table 3). Transcripts encoding matrix proteins that are more abundant in adults include chondroadherin (CHAD), cartilage intermediate layer protein (CILP), cartilage oligomeric matrix protein (COMP), fibronectin (FN), frizzled-related protein (FRZB), and procollagen C-endopeptidase enhancer 2 (PCOLCE2) (Table 4). Redundant cDNA probe sets present on the microarray displayed strong concordance.

**Table 3:** Cartilage matrix proteins and modifying enzymes upregulated in neonatal cartilage relative to adult cartilage

**Gene Name**	**Gene Symbol**	**GenBank ID(s)**	**Fold Change (N/A)**	**p-value**
Procollagen, type II alpha 1	COL2A1	CX594994.1	2.77	0.0161
		CX595082.1	2.31	0.0454
		CX595094.1	7.86	0.0037
		CX596509.1	3.74	0.0059
		CX597852.1	2.62	0.0503
		CX599228.1	2.70	0.0557
		CX599236.1	2.68	0.0402
		CX600104.1	2.64	0.0546
		CX601257.1	3.43	0.0192
		CX602198.1	2.72	0.0488
		CX603857.1	2.43	0.0758
		CX604122.1	2.93	0.0246
		CX604856.1	2.19	0.0310
		CX605192.1	1.75	0.0055
		CX605350.1	2.68	0.0447
		CX605856.1	8.10	0.0040

Procollagen, type IX	COL9A1	CX602049.1	4.61	0.0029
		CX605273.1	6.70	0.0108
	
	COL9A2	CX597674.1	2.11	0.0304
	
	COL9A3	CX594271.1	1.93	0.0083
		CX603177.1	1.41	0.0889

Lysyl oxidase-like 2	LOXL2	CX600483.1	2.98	0.0051
		CX598127.1	2.52	0.0033

Nidogen-2	NID2	CX596324.1	2.71	0.0003
		CX600869.1	2.31	0.0037

Proline 4-hydroxylase, alpha 1	P4HA1	CX605971.1	2.38	< 0.0001
		CX592418.1	2.23	< 0.0001

Tenascin-C	TNC	CX593372.1	4.81	0.0043
		CX601743.1	2.74	0.0430
		CX601953.1	2.25	0.0715
		CX603922.1	3.09	0.0062
		CX604542.1	2.39	0.0016
		CX605854.1	4.72	0.0054

Gene Name & Gene Symbol: gene identity of best BLAST match homologue to cDNA or plasmid; GenBank ID: Accession number of equine cDNA spotted on the microarray; Fold Change (N/A): Relative Neonate/Adult fold change; p-value: one-sample t-test p-value for probe across the four biological replicates in the total sample set.

**Table 4:** Cartilage matrix proteins and modifying enzymes upregulated in adult cartilage relative to neonatal cartilage

**Gene Name**	**Gene Symbol**	**GenBank ID(s)†**	**Fold Change (A/N)**	**p-value**
Cartilage intermediate layer protein	CILP	CX598636.1	5.96	0.0102
		CX599486.1	7.83	0.0042

Cartilage oligomeric matrix protein	COMP	CX592689.1	2.53	0.0036
		CX592951.1	2.25	0.0014
		CX593277.1	2.09	0.0164
		CX597915.1	2.63	0.0041
		CX600749.1	2.43	0.0035
		CX601142.1	2.29	0.0038

Fibronectin	FN1	CX594011.1	4.65	0.0075
		CX596432.1	3.35	0.0192
		CX598808.1	3.86	0.0313
		CX600436.1	4.18	0.0024
		CX601190.1	4.62	0.0017
		CX602630.1	1.31	0.1717
		peFN840	6.03	0.0013
		peFN-C1	7.24	0.0018

Frizzled-related protein	FRZB	CX597477.1	3.53	0.0159
		CX598716.1	3.82	0.0227
		CX604177.1	2.70	0.0015

Heparan sulfate 3-O-sulfotransferase 3A1	HS3ST3A1	CX595901.1	2.61	0.0061

Matrix metalloproteinase 1 (collagenase)	MMP1	peMMP1	4.61	0.0080

Nexin-1	PN-1	CX604123.1	2.27	0.0071

Procollagen C-endopeptidase enhancer 2	PCOLCE2	CX597547.1	4.63	0.0041
		CX601347.1	2.08	0.0036
		CX604313.1	1.89	0.0013
		CX605197.1	4.64	0.0322
		CX605337.1	4.31	0.0038

Serine proteinase inhibitor, clade A, member 5	SERPINA5	CX598843.1	3.27	0.0038

Sulfatase 1	SULF1	CX596711.1	2.83	0.0043

Tissue inhibitor of metallopeptidase 2	TIMP2	CX605012.1	4.48	0.0050

Gene Name & Gene Symbol: gene identity of best BLAST match homologue to cDNA or plasmid; GenBank ID: Accession number of equine cDNA spotted on the microarray (†: some IDs represent plasmid designation); Fold Change (A/N): Relative Adult/Neonate fold change; p-value: one-sample t-test p-value for probe across the four biological replicates in the total sample set.

### Growth factors

With the exception of transforming growth factor-beta receptor 3 (TGFβR3) and fibroblast growth factor 10 (FGF10), no transcripts encoding growth factors or growth factor receptors showed > 2 fold-difference on the microarray hybridizations (Table 5). Several additional growth factors and growth factor receptors (FGFRL1, GDF5, GDF6, IGF2R, IGFBP5) demonstrated a significant change in steady state mRNA levels (p < 0.01), but with only minimal fold change differences between neonatal and adult samples (Table 5).

**Table 5:** Transcriptional profiles of growth factors present on equine cDNA microarray

**Gene Name**	**Gene Symbol**	**Total Probe Sets**	**Median Fold Difference (N/A)**	**Probe sets with p < 0.01**	**Probe sets with p < 0.05**
Bone morphogenetic protein 2	BMP2	6	0.99	0	1
Bone morphogenetic protein 4	BMP4	1	0.81	0	0
Bone morphogenetic protein 6	BMP6	1	0.79	0	0
Bone morphogenetic protein receptor 1a	BMPR1A	1	0.91	0	0
Bone morphogenetic protein receptor 2	BMPR2	2	0.73	0	0
Connective tissue growth factor	CTGF	1	0.82	0	0
Fibroblast growth factor 2	FGF2	2	0.76	0	2
**Fibroblast growth factor 10**	**FGF10**	**1**	**0.35**	**0**	**1**
Fibroblast growth factor 18	FGF18	1	1.03	0	0
Fibroblast growth factor receptor 1	FGFR1	2	0.97	0	0
Fibroblast growth factor receptor 2	FGFR2	3	0.59	0	2
Fibroblast growth factor receptor 3	FGFR3	2	0.74	0	2
Fibroblast growth factor receptor-like 1	FGFRL1	4	0.84	1	2
Growth differentiation factor 5	GDF5	1	0.90	1	1
Growth differentiation factor 6	GDF6	1	1.07	1	1
Growth differentiation factor 10	GDF10	2	0.74	0	1
Insulin-like growth factor 1	IGF1	1	0.89	0	1
Insulin-like growth factor receptor 1	IGFR1	2	0.86	0	0
Insulin-like growth factor 2 receptor	IGF2R	1	1.12	0	1
Insulin-like growth factor binding protein 4	IGFBP4	1	0.83	0	0
Insulin-like growth factor binding protein 5	IGFBP5	3	1.05	1	1
Insulin-like growth factor binding protein 6	IGFBP6	1	0.78	0	0
Insulin-like growth factor binding protein 7	IGFBP7	3	0.85	0	1
Transforming growth factor β1	TGFβ1	2	1.11	0	0
Transforming growth factor β1-like 1	TGFβ1L1	2	0.98	0	0
Transforming growth factor β2	TGFβ2	5	0.96	0	1
Transforming growth factor β3	TGFβ3	2	0.86	0	0
Transforming growth factor β receptor 1	TGFβR1	1	0.73	0	0
Transforming growth factor β receptor 2	TGFβR2	1	0.70	0	0
**Transforming growth factor β receptor 3**	**TGFβR3**	**5**	**0.45**	**3**	**5**
Transforming growth factor β receptor associated protein 1	TGFβRAP1	1	0.91	0	0

Gene Name & Gene Symbol: gene identity of best BLAST match homologue to cDNA or plasmid; Total probe sets: Number of probe sets on the microarray representing the gene based on best BLAST match results; Median Fold Difference (N/A): Relative median Neonate/Adult fold change (**bold **represents fold difference > 2); Probe sets with p < 0.01 or 0.05: number of probe sets for each gene meeting these criteria by one-sample t-test.

### Quantification of differential gene expression with real-time qPCR

Steady state transcript abundance was measured for potential endogenous control genes B2M, GUSB, PGK1, and RPLP0. Messenger RNA levels for all 4 of these genes were similar (p > 0.05) between neonates and adults by the microarray hybridizations. Stability was also assessed by geNorm VBA applet, and RPLP0 and GUSB were the two most stable endogenous control gene candidates (M = 0.552). As a result, they were selected as the most suitable transcripts for the normalization of the target genes of biological interest further analyzed by RT-qPCR.

**Figure 2:**
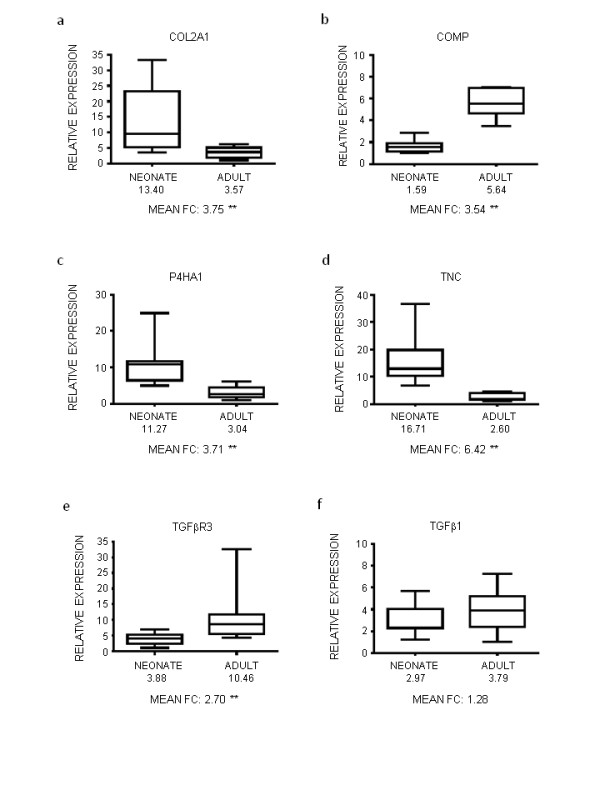
**RT-qPCR measurement of differential gene expression**. Quantification of transcript abundance indicates significant up-regulation of COMP (b) and TGFβR3 (e) gene expression in adult articular cartilage relative to neonatal articular cartilage. In contrast, gene expression for COL2A1 (a), P4HA1 (c), and TNC (d) are significantly up-regulated in neonatal articular cartilage relative to adult articular cartilage. Expression of TGFβ1 (f) was not significantly different between neonatal and adult samples. The RT-qPCR data for all six target genes confirm the results of microarray hybridization experiments. Data are presented as box and whisker plots indicating lowest value, first (0.25) quartile, median, third quartile (0.75), and highest value. Mathematical means of expression are indicated below each age group, and mean fold differences for each target gene are also given numerically as "Mean FC" under the abscissa. ** (p < 0.01).

Five mRNA transcripts (COL2A1, COMP, P4HA1, TGFβR3, TNC) that displayed differential expression in the microarray hybridizations and one transcript (TGFβ1) that did not were selected for RT-qPCR validation (Figure 2). These genes were selected to confirm the microarray findings and because of the functional annotation of their encoded proteins. Relative quantification of target transcripts revealed significant increases in neonatal mRNA abundance for COL2A1, P4HA1, and TNC (Figure 2). Fold change differences were similar or slightly greater than what was measured by microarray profiles. Moreover, increased COMP and TGFβR3 transcript abundance for adult cartilage was also validated by RT-qPCR while TGFβ1 confirmed the microarray finding of no significant difference (Figure 2).

## Discussion

Transcriptional profiling results indicate that maturation-dependent differences in cellular activity exist between neonatal and adult equine articular chondrocytes. These differences likely reflect age-dependent adaptation that occurs as chondrocytes are acting to meet changing functional needs. Neonatal articular cartilage is in a state of growth and expansion. The chondrocytes are secreting large amounts of extracellular matrix (ECM) in response to the expanding articular surface. This can be seen with the upregulation of collagen types II and IX in neonatal chondrocytes. Microarray hybridization data showing increased abundance of type II collagen transcripts in neonatal articular cartilage relative to adult articular cartilage was validated at 3.75-fold by RT-qPCR (p < 0.01, Figure 2).

In neonatal articular chondrocytes, there is also an upregulation of collagen-modifying enzymes necessary for the assembly of ECM; this includes proline 4-hydroxylase (P4H) and lysyl oxidase. P4H is a catalyst necessary for the modification of proline for collagen triple helix formation [32]. Increased transcript abundance for P4HA1 indicated by microarray in neonatal articular cartilage relative to adult articular cartilage was validated at 3.71-fold by RT-qPCR (p < 0.01, Figure 2). Lysyl oxidases catalyze the cross-linking and condensation of collagen fibrils by oxidizing lysine and hydroxylysine residues to become lysyl and hydroxylysyl aldehydes [33]. The condensation of collagens helps increase tensile strength within cartilage. As such, the upregulation of LOXL2 is likely necessary for adaptation to shear and weight-bearing stresses. When immature bovine articular cartilage is treated with the lysyl oxidase inhibitor β-aminopropionitrile in an *in vitro *model, a reduction of tensile integrity was noted [34]. These findings are consistent with neonatal expression of collagen modifying enzymes in parallel to higher levels of collagen synthesis during growth and biomechanical adaptation.

Increased steady state levels of tenascin-C (TNC) transcripts in neonatal cartilage are also consistent with a remodeling process. This protein is upregulated in tissues at developmental stages, during wound healing, and during tumorigenesis [35-37]. TNC has an anti-adhesive N-terminal domain and a pro-adhesive C-terminal domain; its multiple domains allow for interactions with many ECM components, including fibronectin [38,39]. Thus, the presence of TNC affects cells within the ECM, notably by inhibiting cell adhesion and focal adhesion kinase-signaling while stimulating growth [35,37]. The effects of TNC on matrix organization have been well characterized in the developing chick forebrain, in healing skin wounds, and in tumorigenesis [37,40]. Tenascin-C is expressed within the developing limb; however, it disappears throughout the limb and the growth plate during endochondral ossification, though expression can still be detected in postnatal articular cartilage [38,41]. As maturation occurs in normal articular cartilage and the provisional matrix is replaced by adapted anisogenous matrix, TNC expression decreases [38,41]. The current data are consistent with previous TNC findings for chicken, mouse, and rat. Theories for the postnatal presence and then reduction of TNC within the articular cartilage include maintenance of the chondrocyte phenotype, capability of the cells to attach and then detach in an expanding matrix, and the utilization of signal transduction for mediating chondrocyte developmental fate [38].

Another interesting finding was elevated steady state levels of transcripts encoding nidogen-2 (NID2) in neonatal chondrocytes (Table 3). NID2 is one of the two nidogen matrix-binding isoforms found to exist in mammals which are involved in basement membrane assembly [42-44]. Nidogens are typically localized to basement membranes, but have also been associated with development and tissue remodeling, including limb formation [45,47]. Thus far, it has been shown that nidogens interact with perlecan, collagen types I and IV, integrins, and laminins [44,45,48]. Matrix-binding proteins like nidogens also have the capacity to play a role in the adaptive maturation process of a growing neonate, which seems to be best characterized by the expansion of collagens within the provisional immature matrix and the modifications of the matrix postnatally to withstand stresses.

The spectrum of upregulated transcripts in mature articular cartilage is consistent with chondrocytes that have transitioned functionally from cartilage growth to cartilage maintenance. Besides elevated adult steady state transcript levels for matrix molecules like cartilage intermediate layer protein (CILP), cartilage oligomeric matrix protein (COMP), and fibronectin (FN), the data also reveal other clones that contribute to the roles of chondrocytes in mature cartilage (Tables 2 and 4). With regards to matrix proteins, differences in levels of CILP mRNA detected by microarray hybridization correspond to previous reports of protein accumulation in the extraterritorial matrix of adult cartilage with age [49]. It is believed that this protein causes increased calcium pyrophosphate dihydrate deposition in cartilage, impairs chondrocyte responsive to IGF-1, and subsequently hinders matrix repair [50]. Moreover, increased levels of mRNA encoding COMP were also found in adult cartilage; RT-qPCR quantified this difference as 3.5-fold (Figure 2). COMP is a homopentameric glycoprotein that binds to type II collagen interterritorially to stabilize the ECM [51,52]. COMP mRNA upregulation has been previously described during different stages of limb and joint development with species-dependence as well as with mechanical compression [52,53]. Increased fibronectin mRNA hybridization in adult articular cartilage parallels findings from Burton-Wurster *et al*. (Table 4) [54]. Fibronectin is an important extracellular matrix adhesion glycoprotein for cell-matrix signaling. Besides genes which encode matrix proteins, categories of many other genes which typify the maintenance of a heterogeneous matrix are represented. For example, genes with functional roles in collagen-, glycosaminoglycan-, and proteoglycan-binding are upregulated in adult articular chondrocytes (Table 4). Once cartilage growth and adaptation are complete, type II collagen expression is sustained yet at lower levels as the chondrocyte's primary role transforms to tissue maintenance. Increased mRNA levels of matrix metalloproteinase-1 (MMP1), tissue inhibitor of metallopeptidases-2 (TIMP2), and chondroprotective nexin-1 (PN1) are also consistent with cartilage maintenance (Table 4). With maturation, gene expression in articular chondrocytes reflects tissue homeostasis and a perpetuation of the ability to withstand shear and weight-bearing stresses.

Histological examination of articular cartilage reveals increased cellularity per unit volume of cartilage in neonatal animals compared to adults. A stable chondrocyte number and expanding matrix may be a primary mechanism in achieving the lower cell density observed in adult articular cartilage. In comparing pro- vs. anti- cell proliferation, cell cycle, and apopoptic genes in the transcriptional profiles, questions with regards to cell proliferation and cell cycle regulation persist. From the transcriptional profiles, it is difficult to say definitively at either stage of maturation that the chondrocytes are in a state of proliferation or quiescence. Relatively few probes representing these functional categories were significantly up-regulated in neonates. Moreover, though many more (25) gene probes representing these ontology categories were upregulated in adults, distribution did not favor cell proliferation or growth factor activity. Growth factors such as TGFβ1 and IGF1 have been shown to induce chondrocyte proliferation and ECM production with limb development and in culture, and the importance of factors and receptors like BMPR1A, GDF5, and TGFβR2 in the maintenance of postnatal articular have also been demonstrated [44,55-58]. However, FGF10 and TGFβR3 were the only genes related to growth factor activity with > 2 fold difference between adult and neonatal articular cartilage (Table 5). Increased steady state levels of TGFβR3 in articular cartilage for adults were validated by qPCR at 2.70-fold (p < 0.01). While transcript levels for growth factors were relatively similar, it is possible that growth factor activity in postnatal articular cartilage maturation is regulated primarily at a post-transcriptional level. Growth factors sequestered within the extracellular matrix of articular cartilage have been shown to become functionally activated by enzymes like matrix metalloproteinases [59,60].

A broad disparity existed in the number of differentially expressed genes between neonates and adults (Figure 1c). Of the 544 significant genes represented in the array data, a majority (499 genes, or 91.7%) represented increased steady state transcript levels in adult chondrocytes. This difference could be explained by one of two theories. It is possible that cellular activity is more complex in the adult chondrocyte which is thus represented by an increased abundance of transcripts. A second and perhaps more likely explanation is that the discrepancy is accounted for by an adult-biased representation of probe sets on the microarray. Articular cartilage from the 15-month old horse utilized in the generation of the cDNA library spotted on the array likely reflects an adult articular chondrocyte transcriptome with neonate-specific transcripts under-represented. Thus, it is possible that cell proliferation or cell cycle regulatory genes expressed in the neonate were absent from the array and thus were unable to be assessed. A further opportunity to extend the data from the current study would be to conduct time course experiments comparing transcriptional profiling data from immature and adolescent ages to neonatal and adult samples, while including joint-specific and location-specific comparisons in the articular cartilage maturation assessment [61].

## Conclusion

Previous studies of postnatal articular cartilage maturation in mammals have focused on age-dependent changes in the structural and biochemical organization of the matrix. The current data extends this knowledge by using transcriptional profiling to compare gene expression between neonatal and adult chondrocytes in equine articular cartilage. Seven percent (642) of the 9,367 probe sets on an equine-specific microarray were differentially expressed (p < 0.01), organizing primarily into ontology categories related to extracellular matrix, skeletal development, and cell adhesion. Collagens, matrix-modifying enzymes, and provisional matrix proteins were expressed at higher levels in the articular cartilage of newborn foals. Transcripts encoding leucine-rich small proteoglycans, matrix assembly, and cartilage maintenance proteins were found at higher steady state levels in adult articular chondrocytes. Microarray data for COL2A1, COMP, P4HA1, TGFB1, TGFBR3, and TNC were validated by quantitative PCR. Overall, the findings support the hypothesis that gene expression changes in articular cartilage during postnatal maturation reflect a functional transition of chondrocytes from growth and matrix synthesis in the neonate to cartilage homeostasis in the adult.

## Competing interests

The authors declare that they have no competing interests.

## Authors' contributions

MJM and JNM carried out the sample collections and molecular biology studies. LH and AJS carried out the statistical analyses for microarray studies. MJM performed statistical analyses for validation studies. All authors contributed to the writing of this manuscript. All authors read and approved the final manuscript.

## Pre-publication history

The pre-publication history for this paper can be accessed here:



## References

[B1] Brama PA, TeKoppele JM, Bank RA, van Weeren PR, Barneveld A (1999). Influence of site and age on biochemical characteristics of the collagen network of equine articular cartilage. Am J Vet Res.

[B2] Brama PA, Tekoppele JM, Bank RA, Barneveld A, van Weeren PR (2002). Development of biochemical heterogeneity of articular cartilage: influences of age and exercise. Equine Vet J.

[B3] Brommer H, Brama PA, Laasanen MS, Helmimen HJ, van Weeren PR, Jurvelin JS (2005). Functional adaptation of articular cartilage from birth to maturity under the influence of loading: a biochemical analysis. Equine Vet J.

[B4] Hall BK (2005). Bones and Cartilage: Developmental and Evolutionary Skeletal Biology.

[B5] Hunziker EB, Kapfinger E, Geiss J (2007). The structural acrhitecture of adult mammalian articular cartilage evolves by a synchronized process of tissue resorption and neoformation during postnatal development. Osteoarthritis and Cartilage.

[B6] Jadin KD, Bae WC, Schumaker BL, Sah RL (2007). Three-dimensional (3-D) imaging of chondrocytes in articular cartilage: growth-associated changes in cell organization. Biomaterials.

[B7] Brama PA, Tekoppele JM, Bank RA, Barneveld A, van Weeren PR (2000). Functional adaptation of equine articular cartilage: the formation of regional biochemical characteristics up to age one year. Equine Vet J.

[B8] Brama PA, Tekoppele JM, Bank RA, Karssenberg D, Barneveld A, van Weeren PR (2000). Topograpical mapping of biochemical properties of articular cartilage in the equine fetlock joint. Equine Vet J.

[B9] Hoogen BM van den, Lest CH van den, van Weeren PR, van Golde LM, Barneveld A (1999). Effect of exercise on the proteoglycan metabolism of articular cartilage in growing foals. Equine Vet J.

[B10] Sasano Y, Furusawa M, Ohtani H, Mizoguchi I, Takahashi I, Kagayama M (1996). Chondrocytes synthesize type I collagen and accumulate the protein in the matrix during development of the rat tibial articular cartilage. Anat and Embry (Berlin).

[B11] Bland YS, Ashhurst DE (1996). Development and ageing of the articular cartilage of the rabbit knee joint: distribution of fibrillar collagens. Anat and Embry (Berlin).

[B12] Bank RA, Bayliss MT, Lafeber FPJG, Maroudas A, Tekoppele JM (1998). Ageing and zonal variation in post-translational modification of collagen in normal human articular cartilage. Biochemical J.

[B13] Helminen HJ, Hyttinen MM, Lammi MJ, Arokoski JPA, Lapveteläinen T, Jurvelin J, Kiviranta I, Tammi M (2000). Regular joint loading in youth assists in the establishment and strengthening of the collagen network of articular cartilage and contributes to the prevention of osteoarthrosis later in life: a hypothesis. J Bone Min Metab.

[B14] MacLeod JN, Burton-Wurster N, Gu DN, Lust G (1996). Fibronectin mRNA splice variant in articular cartilage lacks bases encoding the V, III-15, and I-10 proteins segments. J Biol Chem.

[B15] MacLeod JN, Fubini SL, Gu DN, Tetreault JW, Todhunter RJ (1998). Effect of synovitis and corticosteroids on transcription of cartilage matrix proteins. Am J Vet Res.

[B16] Chomczynski P, Mackey K (1995). Modification of the TRI Reagent™ procedure for isolation of RNA from polysaccharide- and proteoglycan-rich sources. BioTechniques.

[B17] MacLeod JN (2005). Equine articular cartilage microarray (abstract). Plant & Animal Genome Conference XIII.

[B18] Coleman SJ, Gong G, Gaile DP, Chowdary BP, Bailey E, Liu L, MacLeod JN (2007). Evaluation of COMPASS as a comparative mapping tool for ESTs using horse radiation hybrid maps. Animal Genetics.

[B19] Band MR, Olmstead C, Everts RE, Liu ZL, Lewin HA (2002). A 3800 gene microarray for cattle functional genomics: comparison of gene expression in spleen, placenta, and brain. Animal Biotechnology.

[B20] Eberwine J, Yeh H, Miyashiro K, Cao Y, Nair S, Finnell R, Zettel M, Coleman P (1992). Analysis of gene expression in single live neurons. PNAS USA.

[B21] Feldman AL, Costouros NG, Wang E, Qian M, Marincola FM, Alexander HR, Libutti SK (2002). Advantages of mRNA amplification for microarray analysis. BioTechniques.

[B22] Rosenzweig BA, Pine PS, Domon OE, Morris SM, Chen JJ, Sistare FD (2004). Dye-bias correction in dual-labeled cDNA microarray gene expression measurements. Env Health Persp.

[B23] Coleman SJ, Clinton R, MacLeod JN (2007). Construction of a master gene list for a 9322 feature equine cDNA microarray (P587). Abstracts of Plant & Animal Genome Conference XV.

[B24] Altschul SF, Gish W, Miller W, Myers EW, Lipman DJ (1990). Basic Local Alignment Search Tool. J Mol Biol.

[B25] Dennis G, Sherman BT, Hosack DA, Yang J, Gao W, Lane HC, Lempicki RA (2003). DAVID: Database for Annotation, Visualization, and Integrated Discovery. Genome Biology.

[B26] Maglott D, Ostell J, Pruitt KD, Tatusova T (2005). Entrez Gene: gene-centered information at NCBI. Nucleic Acids Research.

[B27] Hosack DA, Dennis G, Sherman BT, Lane HC, Lempicki RA (2003). Identifying biological themes within lists of genes with EASE. Genome Biology.

[B28] Barrett T, Troup DB, Wilhite SE, Ledoux P, Rudnev D, Evangelista C, Kim IF, Soboleva A, Tomashevsky M, Edgar R (2007). NCBI GEO: mining tens of millions of expression profiles database and tools update. Nucleic Acids Research.

[B29] Ramakers C, Ruijter JM, Lekanne Deprez RH, Moorman AFM (2003). Assumption-free analysis of quantitative real-time PCR data. Neuroscience Letters.

[B30] Schefe JH, Lehmann KE, Buschmann IR, Unger T, Funke-Kaiser K (2006). Quantitative real-time RT-PCR data analysis: current concepts and the novel "gene expression's C_T _difference" formula. J Mol Med.

[B31] Vandesompele J, De Preter K, Pattyn F, Poppe B, Van Roy N, De Paepe A, Speleman F (2002). Accurate normalization of real-time quantitative RT-PCR data by geometric averaging of multiple internal control genes. Genome Biology.

[B32] Myllyharju J, Kivirikko KI (2004). Collagens, modifying enzymes and their mutations in humans, flies, and worms. Trends in Genetics.

[B33] Lucero HA, Kagan HM (2006). Lysyl oxidase: an oxidative enzyme and effector of cell function. Cellular & Molecular Life Science.

[B34] Asanbaeva A, Masuda K, Thonar JMA, Klisch SM, Sah RL (2008). Cartilage growth and remodeling: modulation of balance between proteoglycan and collagen network *in vitro *with β-aminopropionitrile. Osteoarthritis and Cartilage.

[B35] Chiquet-Ehrismann R, Tucker RP (2004). Connective tissues: signaling by tenascins. Int J Biochem & Cell Biol.

[B36] Hsai HC, Schwarzbauer JE (2005). Meet the tenascins: multifunctional and mysterious. J Biochem.

[B37] Mackie EJ, Tucker RP (1999). The tenascin-C knockout mouse revisited. J Cell Sci.

[B38] Pacifici M, Iwamoto M, Golden EB, Leatherman JL, Lee YS, Chuong CM (1993). Tenascin is associated with articular cartilage development. Dev Dyn.

[B39] Pas J, Wyszko E, Rolle K, Rychlewski L, Nowak S, Zukiel R, Barciszewski J (2006). Analysis of structure and function of Tenascin-C. Int J Biochem & Cell Biol.

[B40] Metzger M, Bartsch S, Bartsch U, Bock J, Schachner M, Braun K (2006). Regional and cellular distribution of the extracellular matrix protein tenascin-C in the chick forebrain and its role in neonatal learning. Neuroscience.

[B41] Mackie EJ, Ramsey S (1996). Expression of tenascin in joint-associated tissues during development and postnatal growth. J Anat.

[B42] Carlin B, Jaffe R, Bender B, Chung AE (1981). Entactin, a novel basal lamina-associated sulfated glycoprotein. J Biol Chem.

[B43] Kimura N, Toyoshima T, Kojima T, Shimane M (1998). Enactin-2: a new member of basement membrane protein with high homology to enactin/nidogen. Exp Cell Res.

[B44] Kohfeldt E, Sasaki T, Göhring W, Timpl R (1998). Nidogen-2: a new basement membrane protein with diverse binding properties. J Mol Biol.

[B45] Ekblom P, Ekblom M, Fecker L, Klein G, Zhang H, Kadoya Y, Chu M, Mayer U, Timpl R (1994). Role of mesenchymal nidogen for epithelial morphogenesis in vitro. Development.

[B46] Böse K, Nischt R, Page A, Bader BL, Paulsson M, Smyth N (2006). Loss of nidogen-1 and -2 results in syndactyly and changes in limb development. J Biol Chem.

[B47] Nischt R, Schmidt C, Mirancea N, Baranowski A, Mokkapati S, Smyth N, Woenne EC, Stark H, Boukamp P, Breitreutz D (2007). Lack of nidogen-1 and nidogen-2 prevents basement membrane assembly in skin-organotypic coculture. J Inves Dermatol.

[B48] Erickson AC, Couchman JR (2000). Still more complexity in mammalian basement membranes. J Histochem Cytochem.

[B49] Lorenzo P, Bayliss MT, Heinegard D (1998). A novel cartilage protein (CILP) present in the mid-zone of human articular cartilage increases with age. J Biol Chem.

[B50] Johnson K, Farley D, Hu S, Terkeltaub R (2003). One of two chondrocyte-expressed isoforms of cartilage intermediate-layer protein functions as an insulin-like growth factor 1 antagonist. Arth Rheum.

[B51] Salminen H, Perala, Lorenzo P, Saxne T, Heinegard D, Saamanen AM, Vuorio E (2000). Up-regulation of cartilage oligomeric matrix protein at the onset of articular cartilage degeneration in a transgenic mouse model of osteoarthritis. Arth Rheum.

[B52] Koelling S, Clauditz TS, Kaste M, Miosge N (2006). Cartilage oligomeric matrix protein is involved in human limb development and in the pathogenesis of osteoarthritis. Arth Res Ther.

[B53] Giannoni P, Siegrist M, Hunziker EB, Wong M (2003). The mechanosensitivity of cartilage oligomeric protein (COMP). Biorheology.

[B54] Burton-Wurster N, Borden C, Lust G, MacLeod JN (1998). Expression of the (V+C)^- ^fibronectin isoform is tightly linked to the presence of a cartilaginous matrix. Matrix Biology.

[B55] Kawakami Y, Rodriguez-León J, Belmonte JCI (2006). The roles of TGFβs and Sox9 during limb chondrogenesis. Current Opinion in Cell Biology.

[B56] Davies LC, Blain EJ, Gilbert SJ, Caterson B, Duance VC (2008). The potential of IGF-1 and TGFβ1 for promoting "adult" articular cartilage repair: an in vitro study. Tissue Engineering Part A.

[B57] Serra R, Johnson M, Filvaro VEH, LaBorde J, Sheehan DM, Derynck R, Moses HL (1997). Expression of a truncated, kinase-defective TGF-beta type II receptor in mouse skeletal tissue promotes terminal chondrocyte differentiation and osteoarthritis. J Cell Biol.

[B58] Rountree RB, Schoor M, Chen H, Marks ME, Harley V, Mishina Y, Kingsley DM (2004). BMP receptor signalling is required for postnatal maintenance of articular cartilage. PLoS Biol.

[B59] Hildebrand A, Romaris M, Rasmussen LM, Heinegard D, Twardzik DR, Border WA, Ruoslahti E (1994). Interaction of the small interstitial proteoglycans biglycan, decorin and fibromodulin with transforming growth factor β. Biochem J.

[B60] Schneiderman R, Rosenberg N, Hiss J, Lee P, Liu F, Hintz RL, Maroudas A (1995). Concentration and size distribution of insulin-like growth factor-I in human normal and osteoarthritic synovial fluid and cartilage. Arch Biochem Biophys.

[B61] Dik KJ, Enzerink E, van Weeren PR (1999). Radiographic development of osteochondral abnormalities, in the hock and stifle of Dutch Warmblood foals, from age 1 to 11 months. Equine Vet J Suppl.

